# Supplementary material for: End Sequence Analysis Toolkit (ESAT) expands the extractable information from single-cell RNA-seq data
Source: Genome Res. 2016 Oct;26(10):1397–410. doi: 10.1101/gr.207902.116 (PMC5052061; doi:10.1101/gr.207902.116)
Supplement: Supplemental Material [file supp_gr.207902.116_Supp_Materials.tar.gz › ESAT-esat_v0.1/doc/html/dir_07f53aaccb63b7acbf71fb1fed3a7991.html]

ESAT: java/broad/pda/seq/segmentation Directory Reference


ESAT


- Main Page
- Related Pages
- Packages
- Classes
- Files

All Classes Namespaces Files Functions Variables Enumerator Pages

- java
- broad
- pda
- seq
- segmentation

segmentation Directory Reference

Directory dependency graph for segmentation:

|  |  |
| --- | --- |
| Directories | |
| directory | utils |
|  | |

|  |  |
| --- | --- |
| Files | |
| file | AlignmentDataModel.java |
|  | |
| file | AlignmentDataModelStats.java |
|  | |
| file | ContinuousDataAlignmentModel.java |
|  | |
| file | DefineSourceAndSinkFromPairedEnds.java |
|  | |
| file | GeneCounts.java |
|  | |
| file | GenericAlignmentDataModel.java |
|  | |
| file | GeneScore.java |
|  | |
| file | GenomeWithGaps.java |
|  | |
| file | GenomeWithGaps2.java |
|  | |
| file | GlobalSpliceFilter.java |
|  | |
| file | PrecomputeDataAlignmentStats.java |
|  | |
| file | ReadCountNormalizer.java |
|  | |
| file | ReadFilter.java |
|  | |
| file | WrappedIGVIterator.java |
|  | |


---

Generated on Fri Aug 15 2014 15:11:16 for ESAT by  

 1.8.7
